# Predicting 7-Day Survival Using Heart Rate Variability in Hospice Patients with Non-Lung Cancers

**DOI:** 10.1371/journal.pone.0069482

**Published:** 2013-07-23

**Authors:** Jui-Kun Chiang, Terry B. J. Kuo, Chin-Hua Fu, Malcolm Koo

**Affiliations:** 1 Department of Family Medicine, Buddhist Dalin Tzu Chi General Hospital, Chiayi, Taiwan; 2 Institute of Brain Science, National Yang Ming University, Taipei, Taiwan; 3 Department of Neurology, Buddhist Dalin Tzu Chi General Hospital, Chiayi, Taiwan; 4 Medical School, Tzu Chi University, Hualien, Taiwan; 5 Department of Medical Research, Buddhist Dalin Tzu Chi General Hospital, Chiayi, Taiwan; 6 Dalla Lana School of Public Health, University of Toronto, Ontario, Canada; Klinikum rechts der Isar der TU München, Germany

## Abstract

**Background:**

A simple and accurate survival prediction tool can facilitate decision making processes for hospice patients with advanced cancers. The objectives of this study were to explore the association of cardiac autonomic functions and survival in patients with advanced cancer and to evaluate the prognostic value of heart rate variability (HRV) in 7-day survival prediction.

**Methods:**

A prospective study was conducted on 138 patients with advanced cancer recruited from the hospice ward of a regional hospital in southern Taiwan. Information on functional status and symptom burden of the patients was recorded. Frequency-domain HRV was obtained for the evaluation of cardiac autonomic functions at admission. The end point of the study was defined as the survival status at day 7 after admission to the hospice ward. Multivariate logistic regression analyses were performed to evaluate the independent associations between HRV indices and survival of 7 days or less.

**Results:**

The median survival time of the patients was 20 days (95% CI, 17–28 days). Results from the multivariate logistic regression analysis indicated that the natural logarithm-transformed high-frequency power (lnHFP) of a value less than 2 (OR = 3.8, p = 0.008) and ECOG performance status of 3 or 4 (OR = 3.4, p = 0.023) were significantly associated with a higher risk of survival of 7 days or less. Receiver operating characteristic (ROC) curve analysis revealed that the area under the curve was 0.71 (95% CI, 0.61–0.81).

**Conclusions:**

In hospice patients with non-lung cancers, an lnHPF value below 2 at hospice admission was significantly associated with survival of 7 days or less. HRV might be used as a non-invasive and objective tool to facilitate medical decision making by improving the accuracy in survival prediction.

## Introduction

It is well documented that clinicians are overly optimistic in estimating survival prognosis [1], [2]. A systematic review concluded that an accurate survival prediction within one week occurred in only 25% of cases [3]. Survival prediction scales for advanced cancer patients are typically composed of a combination of clinical symptoms and signs, laboratory data, and physicians' estimation. However, the levels of experience in physicians and the length of patient-physician relationship may affect the accuracy of physician estimation [4], [5]. Such potential bias may be minimized if survival predictions can be made based on more objective measurements.

Heart rate, measured as beat-to-beat intervals, varies in time and this phenomenon is known as heart rate variability (HRV) [6]. HRV reflects the status of the autonomic nervous system and can serve as signal markers for various physiological or pathological events such as patient outcome in intensive care unit [7], unplanned readmission for geriatric patients [8], infections in critically ill patients [9], risk of myocardial infarction [10], and development of hypertension in normotensive men [11].

Recently, the clinical utility of HRV for survival prediction in patients with advanced cancer has been evaluated. A study conducted in 71 hospice patients in Korea reported that decreased HRV, defined as standard deviation of normal to normal beat interval (SDNN) of 21.3 milliseconds or less was significantly associated with a shorter length of survival. Other dichotomized frequency domain indices of HRV including total power (TP), low-frequency power (LFP), and high-frequency power (HFP) were not associated with length of survival [12]. In another study of 47 male patients with advanced cancer, strong association between the time domain measure SDNN and survival was observed. Again, no significant associations were found between survival and the frequency domain measures including LFP, HFP, and very-low-frequency power (VLFP) [13].

In our previous study, we reported that survival time was significantly associated with natural logarithm-transformed TP (lnTP) and natural logarithm-transformed HFP (lnHFP) in patients with hepatocellular cancer [14]. In the present study, we extended the use of HRV measurement to patients with non-lung cancers and evaluate the prognostic values of HRV obtained at the time of hospice admission for 7-day survival predictions in patients with advanced cancer.

## Methods

### Ethics Statement

This study was approved by the Human Research Ethics Committee of Buddhist Dalin Tzu Chi General Hospital (No. B09502017) and written informed consent was obtained from patients or their family members. All procedures were undertaken according to the Declaration of Helsinki.

### Study Subjects

A prospective design was used to recruit patients admitted to the hospice ward of the Buddhist Dalin Tzu Chi General Hospital in south Taiwan, from July 2006 to June 2007. During the one-year recruitment period, 430 patients were admitted to the hospice and 350 patients (82%) agreed to take part in the study. Exclusion criteria included patients diagnosed with breast cancer, lung cancer or lung metastasis and those who were on medications that could potentially influence HRV measurements. Of the 150 patients enrolled in the study, 12 patients (8%) had missing baseline data. Therefore, the final number of cases included in the analysis was 138 of which 36 had liver cancer, 19 had colon cancer, 14 had stomach cancer, 20 had head and neck cancer, 8 had pancreatic cancer, 8 had male genitourinary cancer, 9 had female genitourinary cancer, 4 had esophageal cancer, 26 had unknown or other cancers, and 6 of these patients had two cancers. None of the 138 patients were smokers or alcohol users.

### Clinical and HRV Measurements

Information on functional status and symptom burden of the patients including mean muscle power, cognitive impairment, edema, jaundice, ascites, and Eastern Cooperative Oncology Group (ECOG) performance status was recorded. The ECOG performance status was widely used as a clinical indicator of general functional condition and it is defined as follows: 0 for normal function, 1 for minimal functional impairment, 2 for impairment amounting to spending less than 50% of time in bed, 3 for impairment amounting to spending more than 50% of time in bed, and 4 for being completely bed bound.

Electrocardiography (ECG) recordings were taken in the supine position for five minutes in all patients between 2 to 4 pm within 24 hours of admission. ECG signals were taken by precordial leads and were recorded using a 12 bit analog–digital converter (PCL-818, Advantech, Taiwan) with a sampling rate of 1024 Hz. Each QRS complex was identified. The R point of each valid QRS complex was defined as the time point of each heart beat, and the interval between two R points (R–R interval) was estimated as the interval between the current and latter R points (PPI).

Frequency-domain analysis of PPI was performed using the nonparametric method of fast Fourier transform (FFT). For each time segment (288 seconds, 2048 data points), the algorithm estimated the power spectral density on the basis of FFT. The resulting spectrum was corrected for attenuation resulting from the sampling and Hamming window. The power spectrum was subsequently quantified by integration into frequency-domain indices as total spectrum power (TP), high frequency power (HFP, 0.15–0.4 Hz), and the ratio of lower frequency power to high frequency power (LFP/HFP ratio). The power content of the high frequency component corresponds to respiratory sinus arrhythmia and is modulated solely by the parasympathetic nervous system while the LFP/HFP ratio reflects the sympathovagal balance.

### Data Analysis

Summary data are represented as mean ± standard deviation (SD) for continuous variables, and frequency and percentage for categorical variables. Data transformation using natural logarithm was applied to the HRV indices (TP, HFP, and LFP/HFP) to normalize the data distribution. Survival time was defined as the interval (in days) between admission to the hospice ward and date of death or the end of the study (180 days). The median survival time was estimated using Kaplan-Meier curve. The end point of the study was defined as the survival status at day 7 after admission to the hospice ward. Thus, patients with a survival of 7 days or less would be considered as events while those with a survival beyond 7 days would be considered as non-events.

Independent sample t-test and chi-square were used for comparison of continuous and categorical variables, respectively. Univariate logistic regression analysis was performed to analyze the odds ratio of factors associated with patients with survival of 7 days or less. Separate multivariate logistic regression analyses were performed to evaluate the independent associations between survival of 7 days or less with each of the three HRV indices. First, to simplify the model for clinical use, the HRV indices were dichotomized. Nonparametric smoothing from generalized additive model was applied to determine an optimal cut-off point for the HRV indices, after adjusting for significant covariates. Second, variables from the univariate analysis were evaluated using stepwise variable selection procedure with significance levels for entry (SLE) and stay (SLS) set to 0.15. The final regression model was identified manually by dropping the covariates with *p* value >0.05 one at a time until all regression coefficients were significantly different from 0. Global goodness-of-fit of the fitted logistic regression model was assessed by the Hosmer-Lemeshow test. A non-significant result indicates there is no evidence of lack-of-fit of the model.

Furthermore, the prediction performance of the HRV indices was evaluated using a receiver operating characteristic (ROC) curve. The overall discriminatory ability of the final model in predicting survival of 7 days was shown by the area under the curve (AUC). Sensitivity, specificity, positive predictive value (PPV), and negative predictive value (NPV) were calculated. The optimal cut-off was calculated as the minimum value of the square root of [(1 − sensitivity)^2^+ (1 − specificity)^2^], using the pROC coords function in R with the “closest.topleft” option selected. A plot of the sensitivity (true positive) versus 1 − specificity (false positive) were also constructed. All statistical analyses were performed using R version 2.13.0 (R Foundation for Statistical Computing, Vienna, Austria). Two-sided p values ≤0.05 were considered statistically significant.

## Results

The basic characteristics of the patients are presented in Table 1. Of the 138 patients, 29 (21%) had a survival duration of 7 days or less. The mean age of the 138 patients was 67.6 years with 55% males. The median survival time of the patients was 20 days (95% CI, 17–28 days). Cognitive impairment, presence of ascites, and worse ECOG performance status was significantly associated with survival of less than 7 days. Of the three HRV indices, a lower lnTP was significantly associated with survival of less than 7 days.

**Table 1:** Characteristics of the patients enrolled (N = 138).

Variable		mean ± SD or frequency (%)		p-value
		survival ≤ 7 days	survival >7 days	
		n = 29 (21.0)	n = 109 (79.0)	
**Age (years)**		67.8±11.5	67.3±12.9	0.814
**Sex**				0.394
Male		18 (62)	58 (53)	
Female		11 (38)	51 (47)	
**Functional status and symptom burden**				
Mean muscle power		3.1±1.3	3.6±1.1	0.085
Cognitive impairment		18 (62)	39 (36)	0.011
Edema		15 (52)	58 (53)	0.887
Jaundice		14 (48)	38 (35)	0.185
Ascites		14 (48)	29 (27)	0.025
ECOG PS				0.017
	grade 1 or 2	5 (17)	45 (41)	
	grade 3 or 4	24 (83)	64 (59)	
**Heart rate variability indices**				
lnTP		4.76±1.59	5.49±2.25	0.021
lnHFP		0.92±2.26	2.25±3.07	0.055
ln(LFP/HFP)		−0.33±0.82	−0.18±0.97	0.403

SD: standard deviation, ECOG PS: Eastern Cooperative Oncology Group performance status, lnTP: natural logarithm-transformed total power, lnHFP: natural logarithm-transformed high-frequency power, ln(LFP/HFP): natural logarithm-transformed (lower-frequency power/high frequency power).

Univariate logistic regression analysis indicated that cognitive impairment (p = 0.013), presence of ascites (p = 0.028), and ECOG performance status (score = 3 or 4 versus score = 1 or 2) (p = 0.021) were significantly associated with survival of 7 days or less. On the other hand, lnHFP was inversely associated with survival of 7 days or less (p = 0.021) (Table 2).

**Table 2:** Univariate logistic regression analysis for factors associated with survival of 7 days or less.

Variable	OR (95% CI)	p-value
**Age (years)**	1.00 (0.97–1.04)	0.947
**Sex - male vs. female**	1.44 (0.63–3.41)	0.395
**Functional status and symptom burden**		
Mean muscle power	0.70 (0.48–1.00)	0.051
Cognitive impairment	2.94 (1.28–7.03)	0.013
Edema	0.94 (0.41–2.16)	0.887
Jaundice	1.74 (0.76–4.01)	0.188
Ascites	2.57 (1.10–6.02)	0.028
ECOG PS - grade 3 or 4 vs. 1 or 2	3.37 (1.28–10.62)	0.021
**Heart rate variability indices**		
lnTP	0.79 (0.58–1.00)	0.080
lnHFP	0.82 (0.68–0.96)	0.021
ln(LFP/HFP)	0.80 (0.51–1.22)	0.308

OR: odds ratio, 95% CI: 95% confidence interval, ECOG PS: Eastern Cooperative Oncology Group performance status, lnTP: natural logarithm-transformed total power, lnHFP: natural logarithm-transformed high-frequency power, ln(LFP/HFP): natural logarithm-transformed (lower-frequency power/high frequency power).

To further simplify our prediction model for use in clinical settings, we used nonparametric smoothing from generalized additive model to determine an optimal cut-off point of 2 for lnHFP, adjusting for ECOG performance status score. Results from the multivariate logistic regression analysis using the dichotomized lnHFP variable are presented in Table 3. lnHFP of less than 2 was associated with a significantly higher risk of survival of 7 days or less (OR = 3.8, p = 0.008). A worse ECOG performance status (3 or 4 versus 1 or 2) was also associated with a significantly higher risk of similar magnitude (OR = 3.4, p = 0.023).

**Table 3:** Multivariate logistic regression analysis of factors associated with survival of 7 days or less.

Variable	OR (95% CI)	p-value
**lnHFP<2**	3.80 (1.49–11.12)	0.008
**ECOG PS –3 or 4 vs. 1 or 2**	3.42 (1.27–10.95)	0.023

OR: odds ratio, 95% CI: 95% confidence interval, lnHFP: natural logarithm-transformed high frequency power, ECOG PS: Eastern Cooperative Oncology Group performance status.

Hosmer-Lemeshow test, p = 0.501.

**Figure 1:**
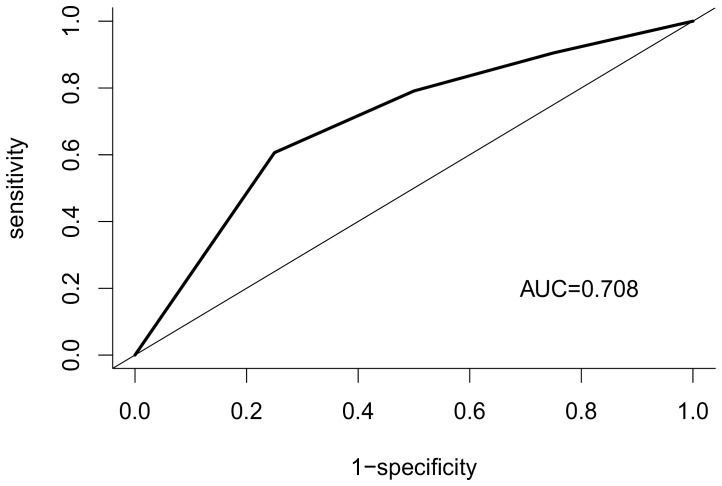
Receiver-operating characteristic curve for the prediction of 7-day survival based on the final model.

The final model, which is based on cut-off values of 2 for lnHFP and grade 3 or 4 for ECOG, yielded a sensitivity of 69% (95% CI, 49–85%), a specificity of 72% (95% CI, 62–80%), a PPV of 39% (95% CI, 26–54%), a NPV of 90% (95% CI, 81–95%). ROC curve analysis revealed an AUC 0.71 (95% CI, 0.61–0.81) for the prediction of 7-day survival (Figure 1).

## Discussion

To our knowledge, this is the first study reporting a significant association between the HRV index lnHFP and 7-day survival in hospice patients with non-lung cancers. The association was independent of the ECOG performance status, which was by itself also a significant prognostic factor in our model. The association between ECOG performance status and survival was as expected. Performance status has consistently been demonstrated as an important clinical factor in estimating survival duration [15]–[18].

Currently, survival prediction scales typically consist of clinical symptoms and signs assessment, laboratory data, and clinician's estimates. Obtaining laboratory measurements from patients with end-stage illness can add to their suffering and therefore, is not universally performed in hospice settings. In addition, HRV measurements are not affected by variability associated with the clinical experience levels of physicians and physician-patient relationship [15]. Hence, there is a clear advantage for the use HRV measurements as a simple, non-invasive, and objective tool for survival prediction in hospice patients with advanced cancer.

Regarding the accuracy of the prediction, Chuang’s Cancer Prognostic Score (CPS), based on eight clinical factors [19] reported an accuracy of 72% and 66% in initial (356 patients) and test set (184 patients), respectively, in predicting survival of less than one week. The accuracy of survival prediction of one week or less in our prediction model, based on only two factors, was 71% and was similar to those obtained by CPS. Thus, our simple prediction model was able to provide comparable levels of accuracy in 7-day survival but without the need for obtaining laboratory measurements.

Furthermore, most existing prediction scales use longer prediction periods than ours. For example, the Palliative Prognostic Index (PPI) is designed for predicting survival in either 3 or 6 weeks [20] and Palliative Prognostic Score Scale (PaP) was developed for 30-day survival prediction [21]. Nevertheless, about one-fifth of our patients did not survive beyond one week. A retrospective descriptive study on 127162 patients in 21 hospice programs across seven states in the United States found that hospice stays of 7 days or less had increased from 26% in 1994 to 34% in 1999 with a median stay decreased from 26 days to 15 days [22]. Similarly, a cohort study using mortality database and claims database in Taiwan found that one-third to a quarter of cancer decedents died within 7 days of hospice enrollment [23]. Therefore, patients with survival of 7 days or less represent a considerable proportion of hospice patients. When a short survival is expected, the goals and plans of management need to be adjusted accordingly. A systematic review of the preferences for place of care and death among patients with advanced cancer revealed that over half of them preferred home care at the end of life [24]. Arrangement of home death in a timely manner is particularly challenging for hospice teams if survival time is short. Therefore, there is a need for tools developed specifically for predicting short hospice stays in order to provide the best end-of-life care to the more severely ill patients.

This study has limitations that are important to note. First, our prediction model might not be applicable for patients with malignant lung cancer or cancers with lung metastasis. HRV is strongly influenced by breathing patterns and are likely to be altered in patients with chest-involved cancers including breast cancer, lung cancer, and cancers with lung metastasis. Therefore, we excluded patients with those conditions in this study. In addition, the interpretation of HRV data in patients with cardiac arrhythmia or after major cardiac surgery is often difficult, which limited the application of HRV in survival prediction in these individuals.

### Conclusions

The present study demonstrated the association between lnHPF at hospice admission and 7-day survival in patients with non-lung cancers. HRV might be used as a non-invasive and objective tool by clinicians to improve 7-day survival prediction for hospice patients with non-lung cancers.
